# Cardiorespiratory Responses During High-Intensity Interval Training Prescribed by Rating of Perceived Exertion in Patients After Myocardial Infarction Enrolled in Early Outpatient Cardiac Rehabilitation

**DOI:** 10.3389/fcvm.2021.772815

**Published:** 2022-01-05

**Authors:** Yaoshan Dun, Shane M. Hammer, Joshua R. Smith, Mary C. MacGillivray, Benjamin S. Simmons, Ray W. Squires, Suixin Liu, Thomas P. Olson

**Affiliations:** ^1^Division of Cardiac Rehabilitation, Department of Physical Medicine and Rehabilitation, Xiangya Hospital of Central South University, Changsha, China; ^2^Division of Preventive Cardiology, Department of Cardiovascular Medicine, Mayo Clinic, Rochester, MN, United States; ^3^National Clinical Research Center for Geriatric Disorders, Xiangya Hospital of Central South University, Changsha, China

**Keywords:** high-intensity interval training, cardiac rehabilitation, myocardial infarction, metabolic gas exchange, rating of perceived exertion

## Abstract

**Objective:** We aimed to determine the cardiorespiratory responses during, and adaptations to, high-intensity interval training (HIIT) prescribed using ratings of perceived exertion (RPE) in patients after myocardial infarction (MI) during early outpatient cardiac rehabilitation (CR).

**Methods:** We prospectively recruited 29 MI patients after percutaneous coronary intervention who began CR within 2 weeks after hospital discharge. Eleven patients (seven men; four women; age: 61 ± 11 yrs) who completed ≥24 supervised HIIT sessions with metabolic gas exchange measured during HIIT once weekly for 8 weeks and performed pre- and post- CR cardiopulmonary exercise tests were included in the study. Each HIIT session consisted of 5–8 high-intensity intervals [HIIs, 1-min RPE 14–17 (Borg 6–20 scale)] and low-intensity intervals (LIIs, 4-min RPE < 12). Metabolic gas exchange, heart rate (HR), and blood pressure during HIIT were measured.

**Results:** The mean oxygen uptake ($\dot{\text{V}}\text{O}$_2_) during HIIs across 88 sessions of HIITs [91 (14)% of $\dot{\text{V}}\text{O}$_2peak_, median (interquartile range, IQR)] was significantly higher than the lower limit of target $\dot{\text{V}}\text{O}$_2_ zone (75% of $\dot{\text{V}}\text{O}$_2peak_) recommended for the HII (*p* < 0.001). Exercise intensity during RPE-prescribed HIITs, determined as %$\dot{\text{V}}\text{O}$_2peak_, was highly repeatable with intra-class correlations of 0.95 (95% CI 0.86– 0.99, *p* < 0.001). For cardiorespiratory adaptations from the first to the last session of HIIT, treadmill speed, treadmill grade, treadmill power, $\dot{\text{V}}\text{O}$_2HII_, %$\dot{\text{V}}\text{O}$_2peak_, and V_E_ during HIIs were increased (all *p* < 0.05), while no difference was found for HR, %HR_peak_ and systolic blood pressure (all *p* > 0.05). $\dot{\text{V}}\text{O}$_2peak_ increased by an average of 9% from pre-CR to post-CR. No adverse events occurred.

**Conclusion:** Our results demonstrate that HIIT can be effectively prescribed using RPE in MI patients during early outpatient CR. Participation in RPE-prescribed HIIT increases exercise workload and $\dot{\text{V}}\text{O}$_2_ during exercise training without increased perception of effort or excessive increases in heart rate or blood pressure.

## Introduction

Exercise-based cardiac rehabilitation (CR) is a secondary prevention tool used worldwide to improve physical function and prognosis in patients after myocardial infarction (MI) (1, 2). High-intensity interval training (HIIT) has recently emerged as an alternative or adjunct strategy to traditional moderate-intensity continuous training (3). HIIT involves alternating periods ranging from a few seconds to 4 min of higher intensity exercise [high-intensity intervals, HIIs: 85 to 95% of peak heart rate (HR) corresponding to 75 to 85% of peak oxygen uptake ($\dot{\text{V}}\text{O}$_2_)] with 1 to 4 min of lower intensity exercise (low-intensity intervals, LIIs: <60% of peak HR) during an exercise session (4). HIIT has been shown to result in similar or greater improvements in aerobic capacity and other health outcomes compared to moderate-intensity continuous training (4). However, the relationships between patient safety, perception of effort, and cardiorespiratory responses and adaptations during HIIT sessions in patients after MI have not been reported. Gaps in our understanding of the relationship between effort perception and cardiorespiratory responses limit our ability to provide optimal guidance for prescription, implementation, and safety of HIIT in CR.

The most common metrics to prescribe aerobic exercise intensity during CR include $\dot{\text{V}}\text{O}$_2_, HR, and their derivative indicators such as percentages of predicted/peak HR and $\dot{\text{V}}\text{O}$_2_; reserves of HR and $\dot{\text{V}}\text{O}$_2_; and metabolic equivalents (METs) (5). During outpatient CR, continuous monitoring of $\dot{\text{V}}\text{O}$_2_ is impractical and, while continuous HR monitoring is feasible, the high number of MI patients prescribed rate modulating pharmacotherapy (e.g., beta-blockers) makes HR a highly variable metric for exercise prescription (6). Furthermore, many patients who begin CR have not performed a graded exercise test, and peak HR has not been determined (7). For these patients, prescribing exercise intensity using predicted peak HR as a guide is imprecise.

Ratings of perceived exertion (RPE) are a practical alternative for prescribing exercise intensity and facilitates relative patient autonomy and progression of exercise intensity during CR (4, 8). Our CR program has used RPE, accompanied by continuous HR and periodic blood pressure monitoring, to prescribe exercise intensity for several decades (9, 10). Our CR staff are experienced in instructing patients on the proper use of the 6–20 Borg RPE scale. Patients are carefully instructed on the use of RPE as part of their baseline graded exercise test and during their first supervised exercise session in CR (10). We have previously demonstrated that RPE-prescribed HIIT improves body composition, characteristics of metabolic syndrome, and cardiorespiratory fitness in patients after MI (11, 12). However, the cardiorespiratory responses directly measured with metabolic gas exchange during, and adaptations to HIIT across several exercise sessions in MI patients have not been previously reported.

Therefore, this study aimed to determine the cardiorespiratory responses and adaptations during HIIT exercise sessions prescribed using RPE in patients with MI who participate in early outpatient CR. We hypothesized that: (1) Using RPE to prescribe exercise intensity will effectively elicit a desired HIIT cardiorespiratory response, and (2) RPE-based HIIT will result in an increasing $\dot{\text{V}}\text{O}$_2_ during exercise training across exercise sessions without increased perception of effort or excessive increases in heart rate and blood pressure.

## Methods

### Participants and Study Design

This prospective observational study initially recruited 29 consecutive MI patients with percutaneous coronary intervention who were referred to outpatient CR within 2 weeks of discharge from inpatient care (our traditional time to begin CR) at Mayo Clinic, Rochester, MN, USA from February 1^st^, 2017, to September 30^th^, 2018. Thirteen patients who did not perform a post-CR cardiopulmonary exercise test (CPET), two who refused to wear a metabolic gas collector/mask during exercise training, and three who changed their exercise type from treadmill to recumbent stationary cycle were excluded. Eleven patients [seven men, four women; age: 62 [11] yrs, median (interquartile range, IQR); BMI: 33.0 (7.2) kg/m^2^; the interval between hospital dismissal and the start of CR: 14 [4] days] who completed ≥24 sessions of supervised HIIT on a treadmill with metabolic gas exchange measured during HIIT once per week for eight consecutive weeks and who performed pre and post CR CPET were included. Cardiovascular medications were unchanged during the study period. Participants were free of angina at low exercise intensities, symptomatic arrhythmias, symptomatic heart valve disease, musculoskeletal limitations to exercise training, and significant frailty or weakness (i.e., inability to engage in HIIT). Study procedures were approved by the Institutional Review Board for Research at Mayo Clinic (Rochester, MN, USA; 15-007977) and conformed to the standards set forth by the Declaration of Helsinki. Patients were informed regarding testing procedures and potential risks of participation before providing written, informed consent.

### High-Intensity Interval Training Intervention

Our protocol for HIIT has been routinely applied in CR for more than a decade and was described previously (4, 13). It was designed for routine use by patients who begin CR within 2 weeks of hospital dismissal and start HIIT after 1 week of moderate intensity aerobic exercise. Components of the exercise prescription were **F**requency: set as three sessions per week for eight consecutive weeks. **I**ntensity: HIIs lasting 1 min at an RPE 14–17 [Borg 6–20 RPE scale] followed by 4-min LIIs at RPE < 12, treadmill speed and grade were self-selected by patients themselves to achieve the target RPEs, and the RPE scores were obtained at the end of each interval. **T**ype: a treadmill was used under continuous observation by clinical exercise physiologists. **T**ime: initial time started at 30 min and gradually progressed to 40 min per session; **V**olume: at least 24 sessions of HIIT completed; **P**rogression: the number of HIIs was gradually increased from 5 to 8 throughout the study according to the patients' expectations and the judgment of clinical exercise physiologists.

Patients were carefully instructed in the proper use of the Borg RPE scale during their first supervised exercise session in outpatient CR. All patients performed adaptive exercise training during the 1st week of CR (three sessions) using RPE ratings of 11–13 to facilitate a gradual accommodation to exercise training, ensure the ability to engage in sustained exercise for a minimum of 20 min, and to become accustomed to the use of the RPE scale. Following the gradual adaptation phase (week-1), the patients started HIIT. Each exercise session began with 5–10 min of low-intensity warm-up (RPE 8–10) and ended with a 5-min low-intensity cool down.

### Cardiopulmonary Exercise Testing

CPETs were conducted by clinical exercise physiologists with cardiologist oversight at the beginning and end of CR. The exercise modality and end-test criteria were consistent between pre-and post-CR tests for all patients. Our operation and interpretation procedures for CPET have been described previously (13).

### Metabolic Gas Exchange Measurements During HIIT

Breath-by-breath $\dot{\text{V}}\text{O}$_2_, carbon dioxide production (VCO_2_), breathing frequency (*f*_B_), and tidal volume (V_T_) were measured continuously using a standard cardiorespiratory diagnostic system (Ultima Series 6 CPX™, MGC Diagnostics Corporation, Minnesota, USA) during an RPE-prescribed HIIT sessions once each week. Continuous cardiorespiratory measurements were performed during a total of 88 HIIT exercise sessions. The cardiorespiratory diagnostic system was calibrated for flow and gas concentrations before each session according to the manufacturer's recommendations using a 3-liter syringe and calibration gases of known concentration. To minimize the influence of subsidiary work, and therefore $\dot{\text{V}}\text{O}$_2_ and VCO_2_, patients were instructed to refrain from excessive stabilization (i.e., using handrails) during all exercise sessions. Minute V_E_ was calculated as the product of V_T_ and breathing frequency (*f*_B_). The respiratory exchange ratio (RER) was calculated as the ratio of VCO_2_ to $\dot{\text{V}}\text{O}$_2_. $\dot{\text{V}}\text{O}$_2HII_ and $\dot{\text{V}}\text{O}$_2LII_ were calculated as the highest average $\dot{\text{V}}\text{O}$_2_ of three consecutive breaths during the HIIs and LIIs (i.e., the highest single-breath $\dot{\text{V}}\text{O}$_2_ value and the preceding and following breaths), respectively. Other metabolic gas exchange values (i.e., RER, VCO_2_, V_E_, V_E_/VCO_2_) were determined by averaging values of the final 15-s of HII and LII, respectively, of each HIIT session.

### Heart Rate and Blood Pressure Measurements

During each HIIT session, HR and rhythm were continuously measured *via* electrocardiogram (ECG) telemetry (Q-Tel RMS, Welch Allyn, New York, USA). The HRs at the end of HIIs and LIIs were recorded. Systolic (SBP) and diastolic (DBP) blood pressures were measured *via* manual sphygmomanometer by clinical exercise physiologists at rest and during the final 15-s of HII and LII, respectively, of each HIIT session.

### Sample Size Calculation

As the primary hypothesis of this study is that RPE prescribed exercise can effectively elicit the desired exercise intensity of HIIT (more than 75% $\dot{\text{V}}\text{O}$_2peak_) in patients after MI, the %$\dot{\text{V}}\text{O}$_2peak_ during HIIs (VO_2HII_ / $\dot{\text{V}}\text{O}$_2peak_ × 100), the gold standard of exercise intensity, was set as the primary endpoint. %$\dot{\text{V}}\text{O}$_2peak_ during HIIs was used to calculate the sample size. We applied the repeated measures analysis model of the Power Analysis & Sample Size software, version 15.0 (NCSS, LLC, USA) to calculate the sample size. The main parameters are as follows: mean %$\dot{\text{V}}\text{O}$_2peak_ during HIIs was 75%, eight sessions of HIITs data from consecutive 8 weeks were collected, the mean increase of %$\dot{\text{V}}\text{O}$_2peak_ during HIIs was 10% across sessions, the standard deviation was 6%, autocorrelation was between 0.2 and 0.4, the dropout rate was estimated 20%. To achieve a power (1-β) of 90% with an α of 0.05, 11 participants were required.

### Statistical Analysis

A minimum of five HIIs was performed by all patients during all HIIT sessions. Therefore, regardless of the number of HIIs performed (ranging from 5 to 8), the final five intervals were used to make comparisons among HIIT sessions. A familiarization HIIT session was used to ensure physiological responses were accurately characterized. The familiarization session was excluded from data analyses, and the second HIIT session was categorized as the first HIIT session. A total of 88 sessions of HIIT data (8 × 11) with gas exchange measurements were included in the analysis.

Data are presented as median [IQR] for continuous variables and frequency and percentage for categorical variables. Repeatability analysis of exercise intensity in terms of %$\dot{\text{V}}\text{O}$_2peak_ was performed with intra-class correlation (ICC) (14) using a random-effects model. Exercise workload (ie., treadmill speed, grade and power in watts) and cardiorespiratory variables measured during the HIIT training sessions (i.e., RPE, $\dot{\text{V}}\text{O}$_2_, %$\dot{\text{V}}\text{O}$_2peak_, V_E_, HR, %HR, BP, energy expenditure [EE] per min and per session) were compared within (HIIs vs. LIIs) and between sessions (first vs. last) using repeated-measures analysis of variance (ANOVA). Pre- and post-CR CPET measurements (i.e., $\dot{\text{V}}\text{O}$_2peak_) were compared via Wilcoxon signed-rank test. %$\dot{\text{V}}\text{O}$_2peak_ and %HR_peak_ during HIITs were calculated as ($\dot{\text{V}}\text{O}$_2HIIorLII_/$\dot{\text{V}}\text{O}$_2peak_) × 100 and (HR _HIIorLII_/HR_peak_) × 100, respectively. $\dot{\text{V}}\text{O}$_2peak_ and HR_peak_ values referred to pre-CR CPET. Treadmill power in watts was calculated (Watts = % treadmill grade × treadmill speed in m·min^−1^ × body weight in kg). EE per min was calculated according to the equation: calories = [$\dot{\text{V}}\text{O}$_2_ in ml·kg^−1^·min^−1^ × body weight in kilograms]/200 as described previously (15). EE per session = EE per min × exercise time. Analyses were performed with SPSS 19.0 (SPSS, Inc). Statistical significance was set at *p* < 0.05.

## Results

Patients' demographic and clinical characteristics are shown in Table 1. Among the 11 patients, four suffered ST-segment elevation MI, and seven suffered non-ST-segment elevation MI; two patients underwent double-vessel PCI, and nine performed single-vessel PCI. The interval between the hospital dismissal and the start of outpatient CR was 14 [4] [median (IQR)] days. Pre CR echocardiograms demonstrated normal left ventricular systolic function with left ventricular ejection fractions of 56% [6%] [median (IQR)]. Echocardiography was not repeated after CR.

**Table 1:** Demographics and clinical characteristics.

*n*	11
Age (years)	62 [11]
Men	7 (64)
Body weight (kg)	98.1 [22.6]
Body mass index (kg/m^2^)	33.0 [7.2]
LVEF (%)	56 [8]
Medical history, *n* (%)	
MI	11 (100)
STEMI	4 (36)
NSTEMI	7 (64)
Coronary angiography	11 (100)
One-vessel disease	5 (46)
Two-vessel disease	3 (27)
Three-vessel disease	3 (27)
Previous MI	2 (18)
Hypertension	6 (55)
Dyslipidemia	11 (100)
Smoking history	5 (45)
Medications, *n* (%)	
ACEI/ARBs	3 (27)
Anticoagulants	5 (45)
Antiplatelet agents	11 (100)
Beta-blockers	10 (91)
Calcium channel blockers	3 (27)
Diuretics	2 (18)
Nitrates	1 (9)
Digoxin	1 (9)
Statins	11 (100)
CPET parameters	
HR_rest_ (bpm)	70 [18]
HR_peak_ (bpm)	141 [54]
SBP_rest_ (mmHg)	123 [30]
DBP_rest_ (mmHg)	71 [16]
SBP_peak_ (mmHg)	180 [24]
DBP_peak_ (mmHg)	76 [20]
Respiratory exchange ratio	1.16 [0.11]
$\dot{\text{V}}\text{O}$_2peak_ (L·min^−1^)	2.4 [0.6]
$\dot{\text{V}}\text{O}$_2peak_ (ml·kg^−1^·min^−1^)	24.0 [6.5]
Number of completed CR sessions	35 [1]
Days between hospital discharge and CR start	14 [4]

*Data presented as median [interquartile range, IQR] for continuous variables or n (%) for categorical variables. SBP, systolic blood pressure; DBP, diastolic blood pressure; LVEF, left ventricular ejection fraction; MI, myocardial infarction; STEMI, ST-segment elevation myocardial infarction; NSTEMI, non-ST segment elevation myocardial infarction; ACEIs, angiotensin-converting enzyme inhibitors; ARBs, angiotensin II receptor blockers; CPET, cardiopulmonary exercise testing; HR, heart rate; CR, cardiac rehabilitation; $\dot{\text{V}}\text{O}$_2peak_, peak oxygen uptake*.

**Figure 1:**
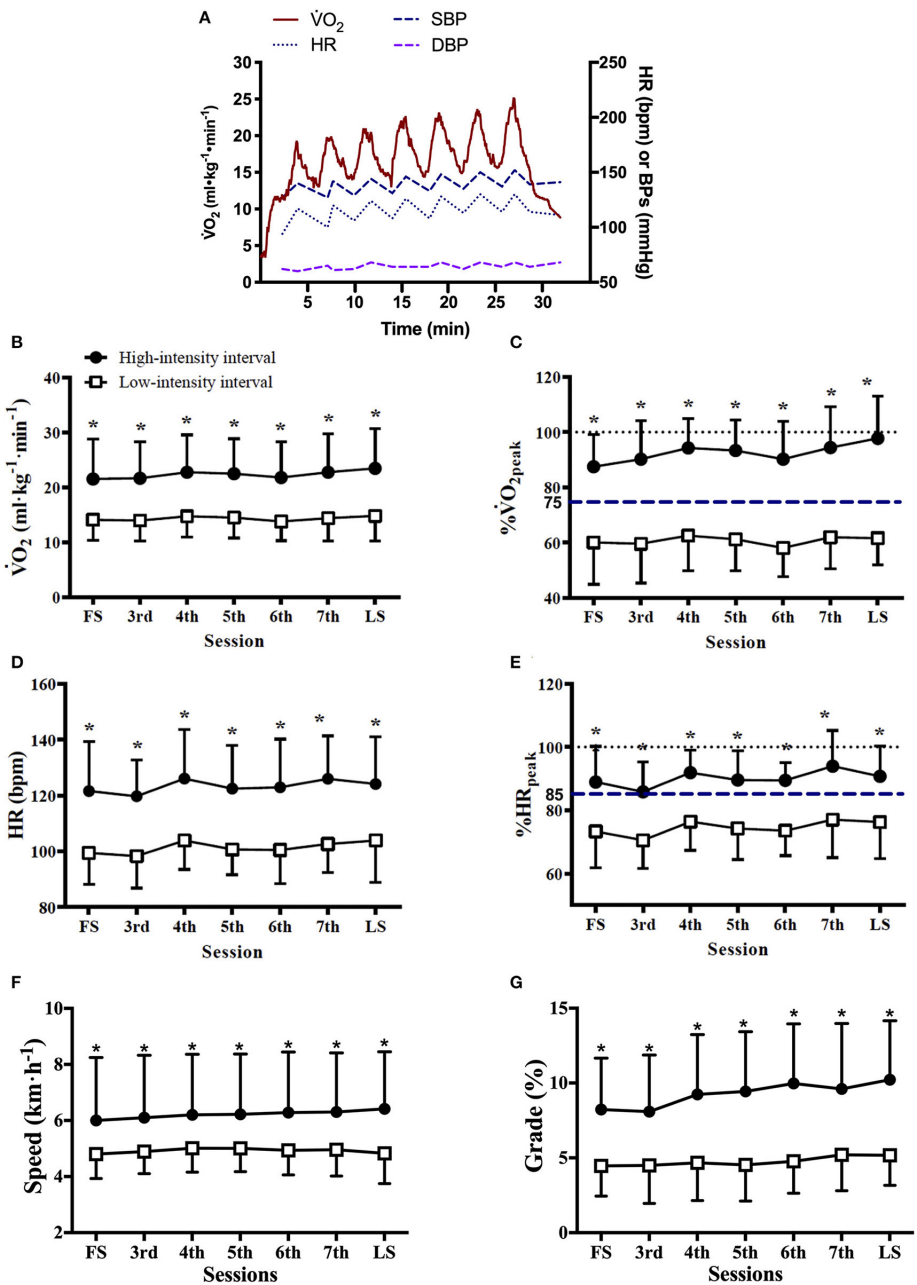
Cardiorespiratory responses and treadmill workload during RPE-prescribed HIIT. **(A)**, a representative patient's oxygen uptake ($\dot{\text{V}}\text{O}$_2_), heart rate (HR), systolic (SBP), and diastolic (DBP) blood pressure responses during a HIIT session. The average $\dot{\text{V}}\text{O}$_2_ **(B)**, %$\dot{\text{V}}\text{O}$_2peak_ **(C)**, HR **(D)**, %HR_peak_ **(E)**, treadmill speed **(F)**, and treadmill grade **(G)** responses to the high- and low-intensity intervals over time. FS is the first exercise session. LS is the last exercise session. Repeated-measures ANOVA was used for all assessments. Data were expressed as mean + up limit of 95% confidence interval for high-intensity intervals and mean–low limit of 95% confidence interval for low-intensity intervals in **(B–G)**. *Significantly higher than low-intensity interval, *p* < 0.001.

Exercise workload and cardiorespiratory responses to RPE prescribed HIIT are presented in Figure 1 and Table 2. The highest mean $\dot{\text{V}}\text{O}$_2_ during HIIs of 88 sessions of HIITs [91 (14)% of $\dot{\text{V}}\text{O}$_2peak_, median (IQR)] was significantly higher than for the target $\dot{\text{V}}\text{O}$_2_ (75% of $\dot{\text{V}}\text{O}$_2peak_) recommended for HIIs (*p* < 0.001). The ICC of exercise intensity, %$\dot{\text{V}}\text{O}$_2peak_, between the RPE-prescribed HIIT sessions was 0.95 (95% CI, 0.86 to 0.99, *p* < 0.001). The values of treadmill speed, treadmill grade, power, $\dot{\text{V}}\text{O}$_2_, %$\dot{\text{V}}\text{O}$_2peak_, HR, %HR_peak_, SBP, V_E_, V_T_ and *f*_B_ in the HIIs were significantly greater than those in the LIIs during each exercise session (all *p* < 0.01), which was consistent with the values of RPE during the HIIs vs. during the LIIs [15 (2) vs. 11 (2), median (IQR), *p* < 0.001]. A difference of 9–11% between %HR_peak_ and %$\dot{\text{V}}\text{O}$_2peak_ was present for the LIIs and is consistent with conventional wisdom as reported in the literature (16) that %HR_peak_ is greater than %$\dot{\text{V}}\text{O}$_2peak_ at a constant workrate. However, for the HIIs, %HR_peak_ was not higher than %$\dot{\text{V}}\text{O}$_2peak_. For the HIIs of the first HIIT session, median %HR_peak_ and %$\dot{\text{V}}\text{O}$_2peak_ were identical (88%), while for the final session %$\dot{\text{V}}\text{O}$_2peak_ was greater than %HR_peak_ (97% vs. 90%) during HIIs. No differences were found in DBP and RER between HIIs and LIIs (all *p* > 0.05).

**Table 2:** Treadmill workload and cardiorespiratory variables during high- and low-intensity intervals.

	**High-intensity intervals**		**Low-intensity intervals**	
	**First session**	**Last session**	**First session**	**Last session**
Treadmill speed (km per hour)	5.9 [2.4]	6.2 [2.1]^*^^†^	4.9 [1.1]	4.9 [1.5]
Treadmill grade (%)	8.4 [2.7]	10.3 [5.8]^*^^†^	4.9 [3.2]	5.1 [2.9]^†^
Treadmill power (Watts)	807 [573]	1039 [707]^*^^†^	390 [211]	407 [196]
RPE	14 [2]^*^	15 [2]^*^	11 [1]	11 [2]
$\dot{\text{V}}\text{O}$_2_ (ml·kg^−1^·min^−1^)	21.1 [2.8]^*^	23.3 [3.0]^*^^†^	14.6 [4.6]	14.6 [2.5]
%$\dot{\text{V}}\text{O}$_2peak_	88 [11]^*^	97 [17]^*^^†^	61 [15]	61 [11]
EE per minute (kcal min^−1^)	10.4 [1.5]^*^	11.4 [2.0]^*^^†^	7.1 [2.0]	7.2 [1.3]
EE per 30-min session (kcal)^‡^	62.0 [8.8]^*^	68.5 [11.3]^*^^†^	170.4 [45.8]	171.7 [28.6]
RER	0.95 [0.06]	0.98 [0.07]	0.94 [0.07]	0.93 [0.08]
HR (bpm)	124 [23]^*^	126 [26]^*^	99 [17]	101 [23]
%HR_peak_	88 [8]^*^	90 [14]^*^	70 [8]	72 [11]
SBP (mmHg)	156 [18]^*^	148 [26]^*^	136 [16]	137 [16]
DBP (mmHg)	66 [8]	61 [7]	64 [14]	61 [6]
V_E_ (L·min^−1^)	59 [22]^*^	65 [24]^*^^†^	40 [12]	42 [16]
V_T_ (L)	1.8 [0.6]^*^	1.9 [0.5]^*^	1.4 [0.7]	1.4 [0.8]
*f*_B_ (breaths·min^−1^)	34 [8]^*^	36 [6]^*^	30 [5]	30 [6]
V_E_/VCO_2_	32 [5]	33 [6]^*^	30 [3]	31 [4]

*Data presented as median [interquartile range, IQR]. RPE, rating of perceived exertion; HR, heart rate; $\dot{\text{V}}\text{O}$_2_, oxygen uptake; EE, energy expenditure; VCO_2_, carbon dioxide production; RER, respiratory exchange ratio; SBP, systolic blood pressure; DBP, diastolic blood pressure; V_*E*_, ventilation; V_*T*_, tidal volume; f_*B*_, breathing frequency; VCO_2_, carbon dioxide output*.

* *Significantly greater than the low-intensity interval (p < 0.05)*.

† *Significantly different compared to first session (p < 0.05). Repeated-measures ANOVA was used for all evaluations*.

‡ *30-min session included five 1-min high-intensity intervals and five 4-min low-intensity intervals*.

**Figure 2:**
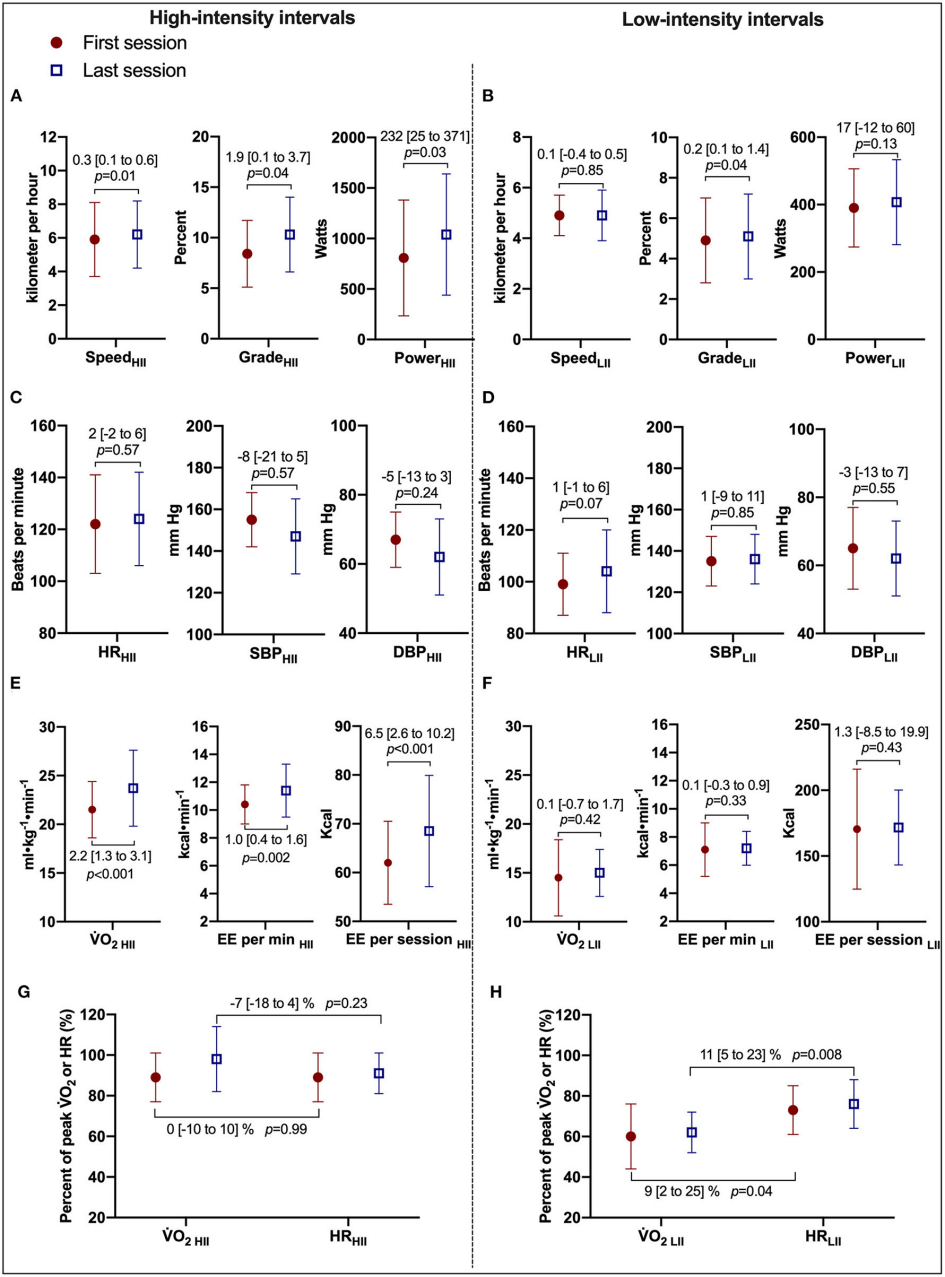
Cardiorespiratory and treadmill workload adaptations to RPE-prescribed HIIT. **(A,B)** Present comparisons of treadmill speed, treadmill grade, and power output between the first and last HIIT sessions during high- and low-intensity intervals, respectively. **(C,D)** Present comparisons of heart rate (HR), systolic (SBP), and diastolic (DBP) blood pressure between the first and last HIIT sessions. **(E,F)** Present comparisons of oxygen uptake ($\dot{\text{V}}\text{O}$_2_), energy expenditure (EE) per minute, and per session. **(G,F)** Present the changes in the relationship of %$\dot{\text{V}}\text{O}$_2peak_ and %HR_peak_ between the first and last HIIT sessions during high- and low-intensity intervals, respectively. HII is high-intensity interval. LII is low-intensity interval. Repeated-measures ANOVA was used for all assessments. was used for all assessments. Data were expressed as mean difference [95% confidence interval].

Comparisons of the first vs. the last exercise sessions to assess cardiorespiratory adaptations during RPE-prescribed HIIT are presented in Table 2 and Figure 2. No differences were found for RPE, HR, %HR_peak_, V_T_, *f*_B_, V_E_/VCO_2_, SBP and DBP (all *p* > 0.05) between the first and last session for both HIIs and LIIs. However, treadmill speed, treadmill grade, power, $\dot{\text{V}}\text{O}$_2_, %$\dot{\text{V}}\text{O}$_2peak_, EE per minute and per session and V_E_ increased significantly from the first to the last session for the HIIs (all *p* < 0.05), while no changes were detected for the LIIs (all *p* > 0.05). No adverse events related to exercise training occurred during the study.

Body mass significantly decreased [98.1 (22.6) kg vs. 95.0 (11.0) kg, median (IQR)] with a mean decrease of 3.1 [95% CI, 0.5 to 5.7] kg (*p* = 0.02). Peak cardiorespiratory variables were determined via CPETs at the beginning and end of CR. $\dot{\text{V}}\text{O}$_2peak_ independent of body mass was not significantly different from pre- to post-CR [2.4 (0.6) L·min^−1^ vs. 2.5 (0.7) L·min^−1^, median (IQR)] with a mean difference of 0.1 [95% CI, −0.1 to 0.3] L·min^−1^ (*p* = 0.21). However, $\dot{\text{V}}\text{O}$_2peak_ dependent on body mass increased in nine of 11 subjects. $\dot{\text{V}}\text{O}$_2peak_ relative to body mass increased [24.0 (6.5) ml·kg^−1^·min^−1^ vs. 26.1 (8.0) ml·kg^−1^·min^−1^, median (IQR)] with a mean increase of 1.9 [95% CI, 0.1 to 3.8] ml·kg^−1^·min^−1^ (*p* = 0.049). In addition, $\dot{\text{V}}\text{O}$_2peak_ as a percentage of age, sex, and anthropometrically predicted values significantly increased from pre- to post-CR [95 (28)% vs. 100 (25)%, median (IQR)] with a mean difference of 5 [95% CI, 1 to 10] % (*p* = 0.04). No additional significant differences were detected in peak exercise cardiorespiratory variables pre-and post-CR.

## Discussion

Our study provided unique metabolic gas exchange data obtained during HIIT sessions and established the efficacy of using self-selected exercise intensity based on a target RPE range as a safe and practical method of prescribing HIIT for patients after MI during early outpatient CR. Our method of prescribing 5–8 one minute HIIs with RPE 14–17 interspersed with 4-min LIIs (RPE < 12) during a 40-min session of training was effective in eliciting a $\dot{\text{V}}\text{O}$_2_ of >95% of pre-training $\dot{\text{V}}\text{O}$_2peak_ during the final HIIT session.

We demonstrated that over 8 weeks of HIIT, patients were able to exercise at a higher $\dot{\text{V}}\text{O}$_2_ without a concurrent increase in RPE or excessive HR and blood pressure response. Patients were able to increase the rate of energy expenditure during the HIIT sessions without an increased perception of effort which is potentially clinically important for decreasing body fat stores with the attendant metabolic health benefits.

Aamot et al. (17) reported that using RPE to prescribe exercise intensity during HIIT resulted in a lower than expected intensity based on %HR_peak_, (detected 82% HR_peak_ vs expected 85% HR_peak_) during HIIs (18) in patients with coronary artery disease in which 80% patients regularly used beta-blockers. In our study, we utilized both %$\dot{\text{V}}\text{O}$_2peak_ (gold standard to reflect exercise intensity) and %HR_peak_ to assess the validity of RPE-prescribed HIIT. Both %$\dot{\text{V}}\text{O}$_2peak_ and %HR_peak_ achieved during HIIs were more than the required levels to meet the designation of high-intensity (75% $\dot{\text{V}}\text{O}$_2peak_ and 85% HR_peak_) for all sessions. $\dot{\text{V}}\text{O}$_2HII_ increased from 88% of $\dot{\text{V}}\text{O}$_2peak_ in the first HIIT session to 97% of $\dot{\text{V}}\text{O}$_2peak_ in the last session.

Patient progression in exercise training dose without the sacrifice of safety is a core tenet of cardiac rehabilitation. We observed that despite an increased $\dot{\text{V}}\text{O}$_2HII_ across exercise sessions, no significant increases in HR, DBP, SBP, or perception of effort were found during RPE-prescribed HIIT. The underlying reasons for this remain unclear. $\dot{\text{V}}\text{O}$_2_ is an integrated indicator of the systems that transport and utilize oxygen, including the respiratory (oxygen uptake from the atmosphere), heart (oxygen transport), peripheral vasculature (oxygen transport, tissue perfusion, tissue diffusion), and skeletal muscle (oxygen extraction and utilization) (19, 20). In the present study, HR, O_2_ pulse [a surrogate for stroke volume (21)], V_E_/VCO_2_ [an indicator of ventilatory efficiency (22)], RER (a variable to reflect degree of exertion) were not significantly changed during 8 weeks of RPE-prescribed HIIT sessions, which suggests that peripheral vasculature and skeletal muscle adaptations may have contributed to the increase in $\dot{\text{V}}\text{O}$_2HII_ across sessions. This hypothesis is supported by our pre-clinical studies in mice that demonstrated regular exercise improved the structure and function of the aortic endothelium (23) and mitochondria in skeletal muscle (24, 25). However, additional research on the mechanisms responsible for these observations is needed.

In order to evaluate the cardiopulmonary adaptations during 8 weeks of HIIT sessions., we studied the relationship between %$\dot{\text{V}}\text{O}$_2peak_ and %HR_peak_ during the HIIT sessions. During LIIs, the values for %$\dot{\text{V}}\text{O}$_2peak_ were 61% for both first and last sessions, and the corresponding %HR_peak_ values were 70 and 72%, respectively. This is consistent with previous reports, where %HR_peak_ was ~10% higher than %$\dot{\text{V}}\text{O}$_2peak_ (26). However, during HIIs, the %$\dot{\text{V}}\text{O}$_2peak_ was 88% for the first session and 97% for the last session, while the corresponding %HR_peak_ remained ~90% for both sessions. This is a clear disconnect from the assumed relationship of %$\dot{\text{V}}\text{O}$_2peak_ and %HR_peak_. Though it has been assumed that the %HR_peak_-%$\dot{\text{V}}\text{O}$_2peak_ relationship holds during HIIT, the expected linear relationship between %$\dot{\text{V}}\text{O}$_2peak_ and %HR_peak_ was established during graded exercise testing with cardiopulmonary measurements and may differ during HIIT. Further studies are needed to elucidate the mechanisms responsible for these observations.

The effect of HIIT on cardiorespiratory fitness in patients with coronary artery disease has been reported, with mean $\dot{\text{V}}\text{O}$_2peak_ increases ranging from 11 to 25% (4). A recent study from our group demonstrated that RPE-prescribed HIIT during early outpatient CR significantly improved $\dot{\text{V}}\text{O}$_2peak_ by 18% (pre-CR vs. post-CR, 23.0 ± 6.3 vs. 28.0 ± 5.9; mean change 5.0 ± 2.5 ml·kg^−1^·min^−1^) in 42 MI patients (12). In the present study, HIIT improved cardiorespiratory fitness (i.e., $\dot{\text{V}}\text{O}$_2peak_) in 9 of 11 (82%) patients, with a mean improvement of only 9%. Possibly related to the small sample size in the current study, statistically significant changes were found in $\dot{\text{V}}\text{O}$_2peak_ related to body mass and % predicted $\dot{\text{V}}\text{O}$_2peak_, but not in $\dot{\text{V}}\text{O}$_2peak_ independent of body mass. The percentage of non-improvement (non-responder: failure to improve $\dot{\text{V}}\text{O}$_2peak_) in CR was 18% in the present study, which is consistent with the data reported in the study by Savage et al. (27) in which 81 out of 385 patients (21%) failed to improve $\dot{\text{V}}\text{O}$_2peak_ during outpatient CR using moderate-intensity continuous training. Our finding of non-improvement in $\dot{\text{V}}\text{O}$_2peak_ with HIIT in some patients is a novel finding. Non-improvement in CR may be associated with exercise intensity, comorbidity score, self-reported physical function, diabetes, and baseline $\dot{\text{V}}\text{O}$_2peak_ (27). In the present study, mean baseline $\dot{\text{V}}\text{O}$_2peak_ was normal and may be a factor in our findings of a less than typical increase in $\dot{\text{V}}\text{O}$_2peak_ and identification of non-responders with HIIT.

The present study did not assess change in left ventricular systolic or diastolic function resulting from HIIT. The literature suggested that HIIT is an effective strategy to attenuate left ventricular remodeling in clinically stable heart failure patients with reduced ejection fraction (28). The effect of HIIT on left ventricular function in heart failure with preserved ejection fraction is controversial (29), while the positive effects of HIIT on exercise capacity and quality of life in patients with MI and heart failure have been reported (4). The patients in the present study were not diagnosed with HFpEF. Further studies are warranted to investigate the effects of HIIT on cardiac function in patients after MI and heart failure with preserved ejection fraction.

## Limitations

Our study examined a single, unique HIIT protocol in MI patients and may not be generalizable to other methods of prescribing HIIT or to other clinical populations. Because measuring metabolic gas exchange data during multiple 30–40 min CR exercise sessions is technically and logistically challenging, we studied only a limited number of patients. Our patients' average baseline $\dot{\text{V}}\text{O}$_2peak_ was in the normal range for healthy individuals and our subjects are not representative of typical post-MI patients. In addition, we did not compare RPE vs. HR-based prescriptions for HIIT. While RPE certainly appears to be an effective prescriptive tool for HIIT, it remains unknown if RPE is the optimal prescription method despite its previously discussed advantages. Additionally, the cardiorespiratory assessments made during HIIT did not include direct measures of cardiac function (e.g., echocardiogram) and relied on an indirect method for cardiac adaptations (i.e., $\dot{\text{V}}\text{O}$_2_ and V_E_/VCO_2_). As such, future studies should consider performing more comprehensive and direct measurements to identify the specific central and peripheral mechanisms responsible for the cardiorespiratory adaptations to RPE-prescribed HIIT in patients after MI.

## Conclusions

RPE is an effective and safe method for prescribing HIIT for patients enrolled in early outpatient CR after uncomplicated MI. Using RPE eliminates reliance on heart rate for exercise intensity prescription and may be advantageous for patients who do not perform a pre-CR exercise test and for individuals receiving heart rate modulating medications. Using an RPE target of 14–17 during 1-min of high-intensity exercise elicits a robust $\dot{\text{V}}\text{O}$_2HII_ of >90% of $\dot{\text{V}}\text{O}$_2peak._ The expected relationship between %HR_peak_ and %$\dot{\text{V}}\text{O}$_2peak_ (%HR_peak_ > %$\dot{\text{V}}\text{O}$_2peak_) is not present during the HIIs of the HIIT. Patients are comfortable performing 5–8 one-minute intervals at >90% of $\dot{\text{V}}\text{O}$_2peak_ during a 40-minute aerobic exercise session. Over the course of eight weeks of HIIT-based CR, patients increased treadmill speed and grade, and $\dot{\text{V}}\text{O}$_2HII_ without an increase in perception of effort or excessive increases in heart rate and blood pressure.

## Data Availability Statement

The raw data supporting the conclusions of this article will be made available by the authors, without undue reservation.

## Ethics Statement

The studies involving human participants were reviewed and approved by Mayo Clinic Institutional Review Board. The patients/participants provided their written informed consent to participate in this study.

## Author Contributions

YD, RS, and TO are responsible for the conception and design of the work. YD, JS, and MM contributed to the acquisition or interpretation of the work. YD, SH, JS, BS, and SL drafted the manuscript. YD, SH, JS, MM, BS, RS, SL, and TO critically revised the manuscript, gave final approval and agree to be accountable for all aspects of the work ensuring integrity and accuracy. All authors have read and approved the final manuscript.

## Funding

The present study was supported by grants from the National Institutes of Health (HL-126638 to TO; T32 HL-07111 to SH and JS; and K12 HD-065987 to JS), National Nature Science Foundation of China (82002403 to YD), Hunan Provincial Nature Science Foundation of China (2021JJ40981 to YD), and the Youth Science Foundation of Xiangya Hospital (2019Q03 to YD).

## Conflict of Interest

The authors declare that the research was conducted in the absence of any commercial or financial relationships that could be construed as a potential conflict of interest.

## Publisher's Note

All claims expressed in this article are solely those of the authors and do not necessarily represent those of their affiliated organizations, or those of the publisher, the editors and the reviewers. Any product that may be evaluated in this article, or claim that may be made by its manufacturer, is not guaranteed or endorsed by the publisher.

## References

[1] Anderson JL Adams CD Antman EM Bridges CR Califf RM Casey DE Jr. 2012 ACCF/AHA focused update incorporated into the ACCF/AHA 2007 guidelines for the management of patients with unstable angina/non-ST-elevation myocardial infarction: a report of the American College of Cardiology Foundation/American Heart Association Task Force on Practice Guidelines. Circulation. (2013) 127:e663–828. 10.1161/CIR.0b013e31828478ac 23630129

[2] O'Gara PT Kushner FG Ascheim DD Casey DE Jr Chung MK de Lemos JA. 2013 ACCF/AHA guideline for the management of ST-elevation myocardial infarction: a report of the American College of Cardiology Foundation/American Heart Association Task Force on Practice Guidelines. Circulation. (2013) 127:e362–425. 10.1161/CIR.0b013e3182742cf6 23247304

[3] Piercy KL Troiano RP Ballard RM Carlson SA Fulton JE Galuska DA. The Physical Activity Guidelines for Americans. JAMA. (2018) 320:2020–8. 10.1001/jama.2018.14854 30418471 PMC9582631

[4] Dun Y Smith JR Liu S Olson TP. High-Intensity Interval Training in Cardiac Rehabilitation. Clin Geriatr Med. (2019) 35:469–87. 10.1016/j.cger.2019.07.011 31543179 PMC6760312

[5] Mezzani A Hamm LF Jones AM McBride PE Moholdt T Stone JA. Aerobic exercise intensity assessment and prescription in cardiac rehabilitation: a joint position statement of the European Association for Cardiovascular Prevention and Rehabilitation, the American Association of Cardiovascular and Pulmonary Rehabilitation and the Canadian Association of Cardiac Rehabilitation. Eur J Prev Cardiol. (2013) 20:442–67. 10.1177/2047487312460484 23104970

[6] Diaz-Buschmann I Jaureguizar KV Calero MJ Aquino RS. Programming exercise intensity in patients on beta-blocker treatment: the importance of choosing an appropriate method. Eur J Prev Cardiol. (2014) 21:1474–80. 10.1177/2047487313500214 23918838

[7] Fletcher GF Ades PA Kligfield P Arena R Balady GJ Bittner VA. Exercise standards for testing and training: a scientific statement from the American Heart Association. Circulation. (2013) 128:873–934. 10.1161/CIR.0b013e31829b5b44 23877260

[8] Iellamo F Manzi V Caminiti G Vitale C Massaro M Cerrito A. Validation of rate of perceived exertion-based exercise training in patients with heart failure: insights from autonomic nervous system adaptations. Int J Cardiol. (2014) 176:394–8. 10.1016/j.ijcard.2014.07.076 25129282

[9] Squires RW Gau GT Miller TD Allison TG Lavie CJ. Cardiovascular rehabilitation: status, 1990. Mayo Clin Proc. (1990) 65:731–55. 10.1016/S0025-6196(12)65134-9 2190053

[10] Squires RW Gau GT. Cardiac rehabilitation and cardiovascular health enhancement. Cardiology: Fundamentals and Practice. Chicago: Year book Medical Publishers (1987). p. 1944–60.

[11] Dun Y Thomas RJ Medina-Inojosa JR Squires RW Huang H Smith JR. High-intensity interval training in cardiac rehabilitation: impact on fat mass in patients with myocardial infarction. Mayo Clin Proc. (2019) 94:1718–30. 10.1016/j.mayocp.2019.04.033 31486378 PMC6755673

[12] Dun Y Thomas RJ Smith JR Medina-Inojosa JR Squires RW Bonikowske AR. High-intensity interval training improves metabolic syndrome and body composition in outpatient cardiac rehabilitation patients with myocardial infarction. Cardiovasc Diabetol. (2019) 18:104. 10.1186/s12933-019-0907-0 31412869 PMC6694483

[13] Skalski J Allison TG Miller TD. The safety of cardiopulmonary exercise testing in a population with high-risk cardiovascular diseases. Circulation. (2012) 126:2465–72. 10.1161/CIRCULATIONAHA.112.110460 23091065

[14] Atkinson G Nevill AM. Statistical methods for assessing measurement error (reliability) in variables relevant to sports medicine. Sports Med. (1998) 26:217–38. 10.2165/00007256-199826040-00002 9820922

[15] Ainsworth BE Haskell WL Herrmann SD Meckes N Bassett DR Jr Tudor-Locke C. 2011 Compendium of Physical Activities: a second update of codes and MET values. Med Sci Sports Exerc. (2011) 43:1575–81. 10.1249/MSS.0b013e31821ece12 21681120

[16] Cunha FA Midgley AW Monteiro WD Farinatti PT. Influence of cardiopulmonary exercise testing protocol and resting VO(2) assessment on %HR(max), %HRR, %VO(2max) and %VO(2)R relationships. Int J Sports Med. (2010) 31:319–26. 10.1055/s-0030-1248283 20200802

[17] Aamot IL Forbord SH Karlsen T Stoylen A. Does rating of perceived exertion result in target exercise intensity during interval training in cardiac rehabilitation? A study of the Borg scale versus a heart rate monitor. J Sci Med Sport. (2014) 17:541–5. 10.1016/j.jsams.2013.07.019 23988787

[18] Karlsen T Aamot IL Haykowsky M Rognmo O. High Intensity Interval Training for Maximizing Health Outcomes. Prog Cardiovasc Dis. (2017) 60:67–77. 10.1016/j.pcad.2017.03.006 28385556

[19] Guazzi M Adams V Conraads V Halle M Mezzani A Vanhees L. EACPR/AHA Scientific Statement. Clinical recommendations for cardiopulmonary exercise testing data assessment in specific patient populations. Circulation. (2012) 126:2261–74. 10.1161/CIR.0b013e31826fb946 22952317 PMC4777325

[20] Guazzi M Arena R Halle M Piepoli MF Myers J Lavie CJ. 2016 focused update: clinical recommendations for cardiopulmonary exercise testing data assessment in specific patient populations. Circulation. (2016) 133:e694–711. 10.1161/CIR.0000000000000406 27143685

[21] Bhambhani Y Norris S Bell G. Prediction of stroke volume from oxygen pulse measurements in untrained and trained men. Can J Appl Physiol. (1994) 19:49–59. 10.1139/h94-003 8186762

[22] Cooper DM Kaplan MR Baumgarten L Weiler-Ravell D Whipp BJ Wasserman K. Coupling of ventilation and CO2 production during exercise in children. Pediatr Res. (1987) 21:568–72. 10.1203/00006450-198706000-00012 3110725

[23] Liu S Zheng F Cai Y Zhang W Dun Y. Effect of Long-Term Exercise Training on lncRNAs Expression in the Vascular Injury of Insulin Resistance. J Cardiovasc Transl Res. (2018) 11:459–69. 10.1007/s12265-018-9830-0 30302742

[24] Dun Y Liu S Zhang W Xie M Qiu L. Exercise combined with rhodiola sacra supplementation improves exercise capacity and ameliorates exhaustive exercise-induced muscle damage through enhancement of mitochondrial quality control. Oxid Med Cell Longev. (2017) 2017:8024857. 10.1155/2017/8024857 29359009 PMC5735688

[25] Xie M Jiang L Dun Y Zhang W Liu S. Trimetazidine combined with exercise improves exercise capacity and anti-fatal stress ability through enhancing mitochondrial quality control. Life Sci. (2019) 224:157–68. 10.1016/j.lfs.2019.03.027 30872179

[26] Riebe D Ehrman JK Liguori G Magal M. ACSM's Guidelines for Exercise Testing and Prescription. Tenth Edition ed Philadelphia: Wolters Kluwer (2016).

[27] Savage PD Antkowiak M Ades PA. Failure to improve cardiopulmonary fitness in cardiac rehabilitation. J Cardiopulm Rehabil Prev. (2009) 29:284–91. 10.1097/HCR.0b013e3181b4c8bd 19935140

[28] Tucker WJ Beaudry RI Liang Y Clark AM Tomczak CR Nelson MD. Meta-analysis of exercise training on left ventricular ejection fraction in heart failure with reduced ejection fraction: a 10-year update. Prog Cardiovasc Dis. (2019) 62:163–71. 10.1016/j.pcad.2018.08.006 30227187 PMC6445773

[29] Mueller S Winzer EB Duvinage A Gevaert AB Edelmann F Haller B. Effect of high-intensity interval training, moderate continuous training, or guideline-based physical activity advice on peak oxygen consumption in patients with heart failure with preserved ejection fraction: a randomized clinical trial. JAMA. (2021) 325:542–51. 10.1001/jama.2020.26812 33560320 PMC7873782

